# Insights Gained from an Approximate Analytical Solution of the Evaporation Model Used by ConsExpo Web

**DOI:** 10.3390/ijerph18062829

**Published:** 2021-03-10

**Authors:** Thomas Schendel, Eva Charlotte Rogasch

**Affiliations:** Exposure, German Federal Institute for Risk Assessment, Max-Dohrn-Straße 8–10, 10589 Berlin, Germany; Eva-Charlotte.Rogasch@bfr.bund.de

**Keywords:** inhalative exposure, exposure modeling, ConsExpo, evaporation, approximate analytical solution, sensitivity analysis

## Abstract

Evaporation of chemicals is an important source of inhalative exposure. We analyzed here the ConsExpo evaporation model, which is characterized by a set of nonlinear differential equations only solvable by numerical means. It shows qualitatively different behavior for different parameters, but the exact conditions remain unclear. This article presents an approximate analytical solution of the ConsExpo evaporation model, derived by using a specific linearization of the nonlinear equations valid for small concentrations. From this solution, three different boundary cases or regimes are found: quick release, near equilibrium, and ventilation driven regime. Depending on the evaporation regime, different parameters influence peak substance air concentration: Quick release regime: total substance amount and room volume; near equilibrium regime: vapor pressure, substance concentration in the product, and molecular weight of the product matrix; ventilation driven regime: vapor pressure, substance concentration in the product, room volume, surface area, mass transfer coefficient, ventilation rate, and molecular weight of the product matrix. A graphical method is developed to display the position of a given scenario in relation to the three regimes. Thus, the approximate analytical solution allows for a given situation to prioritize research for reducing uncertainty of the most sensitive parameters and helps to identify promising risk management measures.

## 1. Introduction

Estimating exposure against chemical substances in products and articles is an important step for risk assessment. Consumer exposure to chemicals can be influenced by various factors, including the product type containing the substance of interest or the variability of consumer use patterns [1,2]. Monitoring consumer exposure can be challenging and time consuming. Mathematical modeling of exposure, on the other hand, is faster, cheaper, and can be applied to large populations [3]. These mathematical models can be relatively simple, such as the approaches used in the European Centre for Ecotoxicology and Toxicology of Chemicals Targeted Risk Assessment (ECETOC TRA) tool [4], or they can be complex, such as the multi chamber near-field far-field air concentration model used in the Consumer Exposure Model (CEM) [5]. This paper focuses on the ConsExpo Web tool [6], which is recommended by the ECHA (European Chemicals Agency) guidance document [7] and which contains several models for different pathways and different degrees of complexity. Since one of the most important exposure routes for chemicals is the entry through the respiratory system via inhalation [8], this paper concentrates on inhalative exposure, more precisely on inhalative exposure caused by evaporation.

ConsExpo was developed for consumer exposure indoors and offers for evaporation three different models. The most sophisticated one, called “Exposure to vapor: Evaporation”, is a mass-balance model, giving a mathematical description of the mass fluxes occurring during the evaporation process [6]. It is based on the two-layer approach developed by Liss and Slater [9] and further refined by Jayjock [10]. It contains an emitting source, backpressure via substance concentration in the air, and removal of the substance due to ventilation. The model has also been extended using Raoult’s law to include the evaporation of a substance, which is but one component of a mixture. It describes the evaporation process via two nonlinear differential equations. Since these differential equations cannot be solved analytically, ConsExpo uses a numerical approach.

The model requires a substantial number of input parameters. Many of these parameters are not substance specific, but depend on the use, such as product amount, treated surface area, or application and exposure time. For the toolbox ConsExpo Web, such information is collected in so called “fact sheets” (https://www.rivm.nl/en/consexpo/fact-sheets (accessed on 26 June 2020)), e.g., the cleaning products fact sheet [11] or the painting products fact sheet [12]. However, the data basis is often weak and needs to be supplemented by expert judgement. Therefore, significant uncertainty can be expected and may affect the outcome of the exposure assessment. A concrete example for a parameter with large uncertainties is the mass transfer coefficient. It describes the diffusion of the substance through the liquid/air boundary layer [13]. A lot of theoretical considerations and experimental measurements have taken place to determine the value of the mass transfer coefficient [13,14,15,16]. A good overview is given in [17] and also in [18]. Nevertheless, the value is often not known, since it is not only substance-specific, but also depends on environmental conditions. Different models predict a range of 2–16 m/h for the value of the mass transfer coefficient [11,19], and as a default ConsExpo uses the value of 10 m/h [18].

To study the impact of the parameter uncertainty, typically a sensitivity analysis is used. Sporadically, such sensitivity analyses have been carried out. For example, Jayjock [10] considered the case of pure substance and unlimited product supply. He used in total four different set of parameters, utilizing for surface area and ventilation rate a small and a large value each. From this, he concluded the necessary conditions under which changes in the ventilation rate actually affect the peak concentration in the room. Moreover, for a simpler evaporation model of ConsExpo (instantaneous release, which does not consider an actual evaporation rate), a sensitivity analysis was carried out by obtaining standardized regression coefficients from Monte Carlo simulations [20]. Four different scenarios with two different substances were used to identify important parameters. However, for the full evaporation model of ConsExpo Web, a comprehensive sensitivity analysis is still missing.

Since the model dynamics can exhibit qualitatively different behavior of substance air concentration, the use of sophisticated methods of sensitivity analysis like variance based sensitivity analysis [21] might be advisable. This method allows for each parameter to define the associated uncertainty and provides its respective contribution to overall variance of the output. However, it should be noted that the results of such an analysis are either specific to a given kind of parameter set, with some parameters remaining constant, and therefore cannot be easily generalized, or that all parameters are varied, including substance specific parameters, and therefore no definitive prediction can be made for a specific substance. Different analyses for different sets of fixed parameters might offer a way out of this dilemma, but given at least nine different input parameters (room volume, surface area, ventilation rate, molecular weight of product matrix and substance, substance concentration in the product, vapor pressure, product amount, and mass transfer coefficient), the task will at least be very cumbersome. It would be helpful if an analytical solution of the model, even an approximate one, could supplement the results of conventional sensitivity analyses. We expect that such an analytical solution could greatly improve the understanding of the dynamics of the evaporation process and provide a quick overview over the most important parameters given the specific situation.

This paper presents an approximate analytical solution applicable for small concentration ranges of the substance in the product. This solution will prove valuable for understanding the complex behavior of the system and allow the identification of different regimes depending on parameter values that exhibit qualitatively different behavior from each other. The approximate analytical solution will be especially useful to identify the important parameters for each regime. As example, an analysis of the impact of the uncertainty associated with the mass transfer coefficient is conducted, demonstrating the benefit of the developed approach. Finally, a graphical method is introduced that allows mapping each exposure situation in relation to the identified regimes. The benefit, especially for an assessment of many substances concerning the same use, will be shown.

## 2. Material and Methods

### 2.1. The Evaporation Model of ConsExpo Web

ConsExpo Web contains three models dealing with inhalative exposure due to evaporation of a substance from a liquid product into room air. We have chosen the mass balance model, (exposure to vapor: evaporation), which is the most realistic one. The model setting describes that the product containing the substance of interest is constantly applied within a certain time in a defined room. The evaporation of the substance from the product into the room air and its subsequent removal by ventilation is modeled; however, no infiltration from outside is considered. The exposed person may stay in the room after the application has finished.

Mathematically, the evaporation process is defined by the following two differential equations (X denotes the amount of substance in the product, Y denotes the amount of the substance in the room air) [6]:

$$\frac{dX}{dt}=-K\;S\frac{M}{RT}\left(P_{eq}-P_{air}\right)+\frac{A_{tot}}{T_{app}}c_{v};t\leq T_{app}$$

$$\frac{dX}{dt}=-K\;S\frac{M}{RT}\left(P_{eq}-P_{air}\right);t>T_{app}$$

(1) $$\frac{dY}{dt}=K\;S\frac{M}{RT}\left(P_{eq}-P_{air}\right)-Q\;Y.$$

K denotes the mass transfer coefficient, S the surface area, M the molecular weight of the substance, R the universal gas constant, T the temperature in the room, $A_{tot}$ the total amount of product used in the room, $T_{app}$ the time during the product is applied, $c_{v}$ the relative (weight) concentration of the substance in the product (dimensionless), and Q the ventilation rate.

Moreover, $P_{eq}$ denotes the equilibrium vapor pressure of the substance in the product and $P_{air}$ denotes the vapor pressure of the substance in the air (backpressure). For the former, Raoult’s law is used; the latter is approximated using the ideal gas law:

(2) $$P_{eq}=\frac{P_{vap}C_{v}}{C_{v}+C_{r}\frac{M}{M_{r}}}$$

(3) $$P_{air}=\frac{RT}{MV}Y$$

(4) $$c_{r}=1-c_{v}\;.$$

$M_{r}$ denotes the molecular weight of the matrix (product except the substance) and V the room volume.

To find an approximate analytical solution to the described evaporation process, we simplify the problem by disregarding the application time and choose instantaneous application instead. We expect that this assumption will significantly alter the exposure values; however, we expect that disregarding the application time will not qualitatively change the behavior of the evaporation process and the main influencing parameters. To put it another way, understanding how evaporation with instantaneous product application works will help to understand how finite application time may alter the exposure results.

Therefore, we assume that the total product amount is applied at once immediately. Hence, the differential equations (Equation (1)) read ($c_{0}$ denotes the initial relative (weight) concentration of the substance in the product and $X_{0}$ the total amount of substance applied):

$$\frac{dX}{dt}=-K\;S\frac{M}{RT}\left(P_{eq}-P_{air}\right)\;;\;X\left(t=0\right)=X_{0}=c_{0}A_{tot}$$

(5) $$\frac{dY}{dt}=K\;S\frac{M}{RT}\left(P_{eq}-P_{air}\right)-Q\;Y.\;$$

The complexity of the problem is at first sight not apparent; however, the relative concentration of the substance in the product $c_{v}$ is a nonlinear function of the total substance amount in the product X:

(6) $$c_{v}=\frac{X}{\frac{X_{0}}{c_{0}}-X_{0}+X}.$$

The sum of the amount of the substance in the product, the substance in the room air, and the substance removed by ventilation need to be constant. To take advantage of this conservation principle, a new variable Z is introduced, which describes the amount of the substance outside of the room.

(7) $$\frac{dZ}{dt}=Q\;Y.$$

Now we can substitute X, because

$$X+Y+Z=X_{0}\to X=X_{0}-Y-Z.$$

Equation (6) can be rewritten as

(8) $$c_{v}=\frac{X}{\frac{X_{0}}{c_{0}}-X_{0}+X}=\frac{X_{0}-Y-Z}{\frac{X_{0}}{c_{0}}-Y-Z}.$$

The equilibrium vapor pressure of the substance in the product (Equation (2)) results in

(9) $$P_{eq}=\frac{P_{vap}C_{v}}{C_{v}+C_{r}\frac{M}{M_{r}}}=P_{vap}\frac{X_{0}-Y-Z}{X_{0}-Y-Z+\frac{M}{M_{r}}X_{0}\left(\frac{1}{c_{0}}-1\right)}=P_{vap}\frac{1-\frac{Y}{X_{0}}-\frac{Z}{X_{0}}}{1-\frac{Y}{X_{0}}-\frac{Z}{X_{0}}+\frac{M}{M_{r}}\left(\frac{1}{c_{0}}-1\right)}.$$

This term is still nonlinear and prohibits an analytical solution of Equation (5).

### 2.2. Approximate Solution for Small Concentrations

We assume that the initial concentration of the substance in the product is small such that:

(10) $$\frac{M}{M_{r}}\left(\frac{1}{c_{0}}-1\right)\gg 1$$

(11) $$\to P_{eq}\approx P_{vap}\frac{1-\frac{Y}{X_{0}}-\frac{Z}{X_{0}}}{\frac{M}{M_{r}}\left(\frac{1}{c_{0}}-1\right)}.$$

The term “small concentrations” is dependent on the ratio of the molecular weights of substance to matrix and will be used in this sense throughout this article.

The system of differential equations (Equations (5) and (7)) are therefore linearized and can be rewritten as

$$\frac{dY}{dt}=K\;S\;\frac{M_{r}}{RT}\;P_{vap}\frac{1-\frac{Y}{X_{0}}-\frac{Z}{X_{0}}}{\left(\frac{1}{c_{0}}-1\right)}-\frac{K\;S}{V}Y-QY$$

(12) $$\frac{dZ}{dt}=Q\;Y.$$

The detailed steps to derive the analytical solution of Equation (12) are outlined in the Supplementary Materials Part A1. Let the y denote the substance concentration in room air (y = Y/V); then, the following solution is derived:

(13) $$y\left(t\right)=\frac{1}{V}\frac{a}{\lambda_{1}-\lambda_{2}}\left(e^{-\lambda_{2}t}-e^{-\lambda_{1}t}\right),$$

with

(14) $$\lambda_{1,2}=\frac{b}{2}\left(1\pm \sqrt{1-\frac{4\;a\;Q}{X_{0}b^{2}}}\right)$$

(15) $$a=K\;S\frac{M_{r}}{RT}\frac{P_{vap}}{\left(\frac{1}{c_{0}}-1\right)}$$

(16) $$b=\frac{K\;S}{X_{0}}\frac{M_{r}}{RT}\frac{P_{vap}}{\left(\frac{1}{c_{0}}\;-\;1\right)}+\frac{K\;S}{V}+Q.$$

The term a/($\lambda_{2}$ − $\lambda_{1}$) describes an upper boundary for the maximal amount of substance in the room air (since the difference of the two exponential terms cannot be larger than 1), while $\lambda_{1}$ characterizes the rate for increase of the substance in room air and the $\lambda_{2}$ characterizes the rate for the decrease of substance in room air.

The system of differential equations in Equation (12) describes three different dynamics of how the initial increase of substance air concentration is slowed down and reversed: First, the concentration of the substance in the product decreases during the evaporation process; therefore, the evaporation rate also decreases. Second, the vapor pressure of the substance in the air (backpressure) limits the maximum amount of substance in room air. Third, due to ventilation, the substance in room air is removed outside. All of these three mechanisms are described by the term b (Equation (16)).

In the following, we are interested in asymptotic cases (hereinafter referred to “regimes”), where one of these three dynamics dominate the other two. The detailed analytical derivations are presented in the Supplementary Materials Part A1.

#### 2.2.1. Quick Release Regime

For this regime, substance amount is the limiting step ((1/$c_{0}$−1) ≈ 1/$c_{0}$ and $X_{0}$/$c_{0}$ = $A_{tot}$, was used):

$$\frac{K\;S}{X_{0}}\frac{M_{r}}{RT}\frac{P_{vap}}{\left(\frac{1}{c_{0}}-1\right)}\gg \frac{K\;S}{V}+Q$$

(17) $$y\left(t\right)\approx \frac{X_{0}}{V}\left(e^{-\lambda_{2}t}-e^{-\lambda_{1}t}\right)$$

(18) $$\lambda_{1}\approx \frac{K\;S}{X_{0}}\frac{M_{r}}{RT}\frac{P_{vap}}{\left(\frac{1}{c_{0}}-1\right)}\approx \frac{K\;S}{A_{tot}}\frac{M_{r}}{RT}P_{vap}$$

(19) $$\lambda_{2}\approx Q.$$

#### 2.2.2. Near Equilibrium Regime

For this regime, vapor pressure in the air (back pressure) is dominant:

$$\frac{K\;S}{V}\gg \frac{K\;S}{X_{0}}\frac{M_{r}}{RT}\frac{P_{vap}}{\left(\frac{1}{c_{0}}-1\right)}+Q$$

(20) $$y\left(t\right)\approx \frac{M_{r}}{RT}\frac{P_{vap}}{\left(\frac{1}{c_{0}}-1\right)}\left(e^{-\lambda_{2}t}-e^{-\lambda_{1}t}\right)$$

(21) $$\lambda_{1}\approx \frac{K\;S}{V}$$

(22) $$\lambda_{2}\approx \frac{V}{X_{0}}\frac{M_{r}}{RT}\frac{P_{vap}}{\left(\frac{1}{c_{0}}-1\right)}Q\approx \frac{V}{A_{tot}}\frac{M_{r}}{RT}Q\;P_{vap}.$$

#### 2.2.3. Ventilation Driven Regime

For this regime, the removal of substance from room air to the outside via ventilation is dominating:

$$Q\gg \frac{K\;S}{X_{0}}\frac{M_{r}}{RT}\frac{P_{vap}}{\left(\frac{1}{c_{0}}-1\right)}+\frac{K\;S}{V}$$

(23) $$y\left(t\right)\approx \frac{K\;S}{Q\;V}\frac{M_{r}}{RT}\frac{P_{vap}}{\left(\frac{1}{c_{0}}-1\right)}\left(e^{-\lambda_{2}t}-e^{-\lambda_{1}t}\right)$$

(24) $$\lambda_{1}\approx Q$$

(25) $$\lambda_{2}\approx \frac{K\;S}{X_{0}}\frac{M_{r}}{RT}\frac{P_{vap}}{\left(\frac{1}{c_{0}}-1\right)}\approx \frac{K\;S}{A_{tot}}\frac{M_{r}}{RT}P_{vap}.$$

### 2.3. Analytical Solution for Pure Substance

As we have derived an approximate analytical solution for small concentrations, we compare it with the solution for pure substance. The details are given in the Supplementary Materials Part A2.

Let t^end^ be the time until the amount of substance in liquid form has completely evaporated; then, the solution reads:

(26) $$y\left(t\right)=\frac{K\;\frac{S}{V}\frac{M}{RT}P_{vap}}{K\frac{S}{V}+Q}\left(1-e^{-\left(K\frac{S}{V}+Q\right)t}\right);t\leq t^{end}$$

(27) $$y\left(t\right)=\left[\frac{K\;\frac{S}{V}\frac{M}{RT}P_{vap}}{K\frac{S}{V}+Q}\left(1-e^{-\left(K\frac{S}{V}+Q\right)t^{*}}\right)\right]e^{-Q\left(t-t^{end}\right)};t\geq t^{end}.$$

There can be in principle three different periods for room air concentration dynamics: First, the increase in room air concentration; second, the plateau phase, where concentration practically remains constant; third, the decrease of room air concentration after all the substance has evaporated. The second stage does not occur in every case.

We want to mention that for the special case of unlimited substance supply, Equation (26) was published already in [10]. Apart from the case of pure substance, for large concentrations and larger molecular weight of the matrix compared to the substance, such that

$$\frac{M}{M_{r}}\left(\frac{1}{c_{0}}-1\right)\ll 1,$$

the derived analytical solution might be a good approximation until most of the substance has evaporated from the product.

In analogue to the defined asymptotic cases or regimes for the assumption of small concentrations, we show the results of the similar analysis here (details given in the Supplementary Materials Part A).

#### 2.3.1. Quick Release Regime

$$X_{0}\ll \frac{K\;S\frac{M}{RT}P_{vap}}{K\frac{S}{V}+Q}.$$

Maximal room air concentration:

(28) $$y=\frac{X_{0}}{V}.$$

The time t^+^ until the maximum of room concentration is reached is estimated to

(29) $$t^{+}\approx \frac{X_{0}\;R\;T}{K\;S\;M\;P_{vap}}.$$

In this regime, there is no plateau stage.

#### 2.3.2. Near Equilibrium Regime

$$X_{0}\gg \frac{K\;S\frac{M}{RT}P_{vap}}{K\frac{S}{V}+Q}\;\mathrm{and}\;K\frac{S}{V}\gg Q$$

Maximal room air concentration:

(30) $$y\approx \frac{M}{RT}P_{vap}.$$

Time scale for concentration increase in room air:

(31) $$t^{+}\approx \frac{V}{K\;S}.$$

The time the system remains in the plateau stage, $t^{*}$ is estimated to

(32) $$t^{*}\approx \frac{X_{0}\;RT}{Q\;V\;M\;P_{vap}}.$$

In the near equilibrium regime, the maximal room air concentration will reach nearly the saturated vapor pressure; the evaporation rate is very small under such a condition.

#### 2.3.3. Ventilation Driven Regime

$$X_{0}\gg \frac{K\;S\frac{M}{RT}P_{vap}}{K\frac{S}{V}+Q}\;;K\frac{S}{V}\ll Q$$

Maximal room air concentration:

(33) $$y\approx \frac{K\;\frac{S}{V}\frac{M}{RT}P_{vap}}{Q}.$$

Time scale for concentration increase in room air:

(34) $$t^{+}\approx \frac{1}{Q}.$$

The time the system remains in the plateau stage is given by:

(35) $$t^{*}\approx \frac{X_{0}\;RT}{K\;S\;M\;P_{vap}}.$$

In the ventilation driven regime, the maximal air concentration will be much lower than the saturated vapor pressure; the evaporation rate is therefore substantial.

### 2.4. Alternative Approximate Analytical Solution

To study the quality of the approximate analytical solution for small concentrations in more detail, we return to the term that was simplified (Equations (9) and (11)):

(36) $$P_{vap}\frac{1-\frac{Y}{X_{0}}-\frac{Z}{X_{0}}}{1-\frac{Y}{X_{0}}-\frac{Z}{X_{0}}+\frac{M}{M_{r}}\left(\frac{1}{c_{0}}-1\right)}\approx P_{vap}\frac{1-\frac{Y}{X_{0}}-\frac{Z}{X_{0}}}{\frac{M}{M_{r}}\left(\frac{1}{c_{0}}-1\right)}.$$

**Figure 1:**
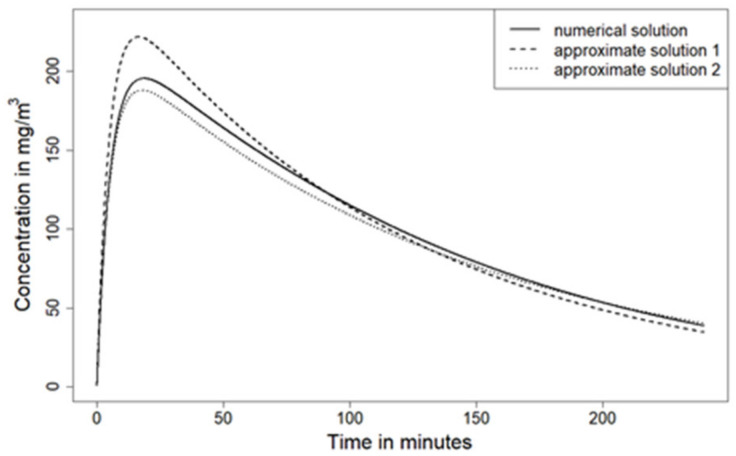
Substance concentration in room air using the numerical solution (solid line), the approximate analytical solution (dashed line, dubbed “approximate solution 1”), and an approximate analytical solution that underestimates the rate of the evaporation process (dotted line, dubbed “approximate solution 2”).

This approximation can be interpreted as a Taylor expansion for the case that nearly all substance has evaporated already, such that 1 − (Y + Z)/$X_{0}$ ≪ 1. The presented approximation leads to a faster evaporation of the substance than the original model, as shown in Figure 1. Therefore, we can construct an analytical solution that will always exhibit a lower evaporation of the substance. Taylor expansion regarding (Y + Z)/$X_{0}$ ≪ 1 (no substantial part of the substance has evaporated yet) leads to

(37) $$P_{vap}\frac{1-\frac{Y}{X_{0}}-\frac{Z}{X_{0}}}{1-\frac{Y}{X_{0}}-\frac{Z}{X_{0}}+\frac{M}{M_{r}}\left(\frac{1}{c_{0}}-1\right)}\approx \frac{P_{vap}}{1+\frac{M}{M_{r}}\left(\frac{1}{c_{0}}-1\right)}-P_{vap}\frac{\frac{M}{M_{r}}\left(\frac{1}{c_{0}}-1\right)\left(\frac{Y}{X_{0}}+\frac{Z}{X_{0}}\right)}{{\left(1+\frac{M}{M_{r}}\left(\frac{1}{c_{0}}-1\right)\right)}^{2}}.$$

Unfortunately, using this approximation leads to continued evaporation, although the substance in the product has already completely evaporated. To fix this unphysical situation, the term involving the substance outside the room and in room air (Z and Y) is altered:

(38) $$P_{vap}\frac{1-\frac{Y}{X_{0}}-\frac{Z}{X_{0}}}{1-\frac{Y}{X_{0}}-\frac{Z}{X_{0}}+\frac{M}{M_{r}}\left(\frac{1}{c_{0}}-1\right)}\approx P_{vap}\frac{1-\frac{Y}{X_{0}}-\frac{Z}{X_{0}}}{1+\frac{M}{M_{r}}\left(\frac{1}{c_{0}}-1\right)}.$$

Comparing this solution with Equation (36), taking into account that for room concentration, the evaporation of the substance into room air is the only source term, and remembering that the set of differential equations has been linearized, it is easy to see that the relative difference between both approximations for room air concentration Δy/y is maximal

(39) $$\left|\frac{\Delta y}{y}\right|\leq \frac{1}{\frac{M}{M_{r}}\left(\frac{1}{c_{0}}-1\right)}.$$

### 2.5. Numerical Solution

The set of differential equations given in Equation (1) is solved by numerical means for given starting conditions. ConsExpo Web (ConsExpo Web. Available online: http://www.consexpoweb.nl (accessed on 19 June 2020)) offers a free available tool. In this paper, we use numerical solutions to compare the goodness of fit of the derived approximate analytical solution. We implemented a numeric scheme using the software R by utilizing a self-written Runge–Kutta algorithm fourth order [22]. The programming code is available in the Supplementary Materials Part D.

## 3. Results

### 3.1. Comparison between Approximate Analytical and Numerical Solution

We have derived an approximate analytical solution for small concentrations:

$$\frac{M}{M_{r}}\left(\frac{1}{c_{0}}-1\right)\gg 1.$$

In Figure 1 (parameters described in the Supplementary Materials Part C), the room concentration of the numerical solution (solid line) and the approximate analytical one (dashed line) are depicted, for example, with

$$\frac{M}{M_{r}}\left(\frac{1}{c_{0}}-1\right)=4.$$

This example was chosen to highlight the differences between numerical and approximate analytical solution for a situation where the assumption is not well met, since four is far from being large against one. Although there are visible deviations in the time course of the room concentration, the qualitative behavior is well captured by the approximate solution. 

An alternative approximate analytical solution that always underestimates the speed of evaporation has been obtained (Equation (38)). This solution is depicted in Figure 1 as a dotted line. The benefit of Equation (38) is that it always underestimates the speed of evaporation (compared with the full ConsExpo Web model), while Equation (36) overestimates it. Therefore, the solution derived from Equation (38) is helpful to assess the magnitude of deviations caused by the approximate analytical solution.

### 3.2. Regimes of Evaporation

**Figure 2:**
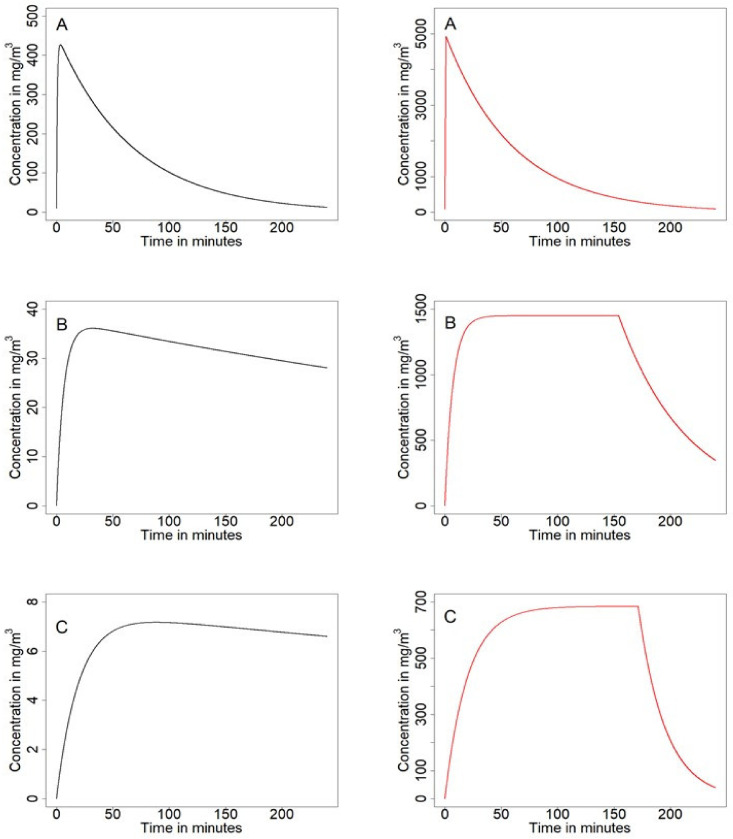
Substance room concentration for small substance concentrations (left) and pure substance (right) for (**A**) the quick release regime; (**B**) the near equilibrium regime; and (**C**) the ventilation driven regime. The according parameters are given in the Supplementary Materials Part C.

For the approximate case of small concentrations and the case of pure substance, we have identified three border regimes: The quick release, the near equilibrium, and the ventilation driven regime. Since these regimes occur in both cases, the approximation for small concentrations and pure substance, they are likely an overall feature of the evaporation process. The time course of air concentration is depicted for all three regimes in Figure 2.

One general difference between the case of small concentrations and the pure substance case is that in the latter one, a plateau stage may occur. For pure substance, the evaporation rate is not influenced by the reduced substance amount until all of it is evaporated, since the substance still amounts to 100% of the product. However, for small initial substance concentration, the evaporation rate decreases, since the concentration of the substance in the product decreases.

The quick release regime occurs if the evaporation term dominates the dynamics, such that neither the backpressure of room air concentration nor the ventilation play much of a role before most or all of the substance has been evaporated from the product. It is characterized by a short time span where nearly all of the substance evaporates and therefore air concentration increases quickly. The decrease of room air concentration is then mediated by the ventilation rate. This regime therefore has a striking resemblance to the instantaneous release model, but is more complex due to the existing time duration needed to reach the maximum air concentration. Peak concentration depends only on substance amount and room volume. This regime has in principle the same qualitative behavior for all concentration ranges. For any given set of parameters (including vapor pressure), the quick release regime occurs if product amount is sufficiently small. Noteworthily, at least for small concentrations, it is not the total substance amount that matters, but the total product amount.

The near equilibrium regime occurs if the backpressure of room air concentration dominates the dynamics. It is characterized by a comparably long period where substance concentration in the product and in room air are nearly in equilibrium, such that actual evaporation is rather low. Time length of this state is indirectly proportional to vapor pressure and ventilation rate. Maximum concentration depends directly linearly on vapor pressure but not on the ventilation rate. This regime occurs for situations where comparably large amount of the product is used and/or the vapor pressure of the substance is low, the treated surface to room volume ratio is large, and the value for the ventilation rate is rather modest.

For the ventilation driven regime, the ventilation rate limits maximal substance air concentration. From a qualitative perspective, the time course of substance air concentration in the near equilibrium regime and ventilation driven regime are hard to distinguish. However, in the ventilation driven regime, the substance concentrations in the product and room air are far from being in equilibrium; hence, substantial evaporation takes place. This leads to much smaller room air concentrations compared to the near equilibrium regime. Interestingly, the formulas for calculating the time scales for increase and decrease of room air concentration are just switched to the case of the quick release regime. This also means that actually the time until the maximum or plateau stage in room air concentration is reached is determined by the ventilation rate. The ventilation driven regime occurs for situations were a comparably large amount of the product is used and/or the vapor pressure of the substance is low, the treated surface to room volume ratio is small, and the value for the ventilation rate is large.

### 3.3. Sensitivity Analysis

Using the analytical solutions for the regimes (Equations (17), (20) and (23)) directly allows the determination of the parameters that actually influence the resulting substance air concentration. In Table 1, the influencing parameters for all regimes are described for both cases: small substance concentration and pure substance, respectively. The dependencies are given for maximal air concentrations, the time scale for air concentration increase, and the time scale for air concentration decrease, or in the case of pure substance, the plateau stage. A (+) indicates that the respective variable depends linearly (and positively) on the chosen parameter, and a (−) that an inverse relationship exists. A small relative change (e.g., 1%) of one parameter will lead to the same relative change in the outcome variable, with opposite direction in case of a (−). We want to stress the point that this is a local sensitivity analysis, which relies on small changes of parameters.

To derive the results of Table 1 for small concentrations, not only the assumption of Equation (10) was used as prerequisite for the derived analytical solution, but also that of (1/$c_{0}$−1) ≈ 1/$c_{0}$ to make simple statements regarding the initial substance concentration in the product. To use the results of Table 1 for small concentrations in a qualitative way, the following inequalities should be met (see Section 3.4, for which circumstances these conditions can be further relaxed):

(40) $$\frac{M}{M_{r}}\left(\frac{1}{c_{0}}-1\right)\geq 2.5$$

(41) $$c_{0}\leq 0.3\;.$$

This ensures that for any (+) or (-) of the initial substance concentration in the product in Table 1 (given the existence of a clear regime), any sufficient small relative change ε will lead to a relative change Δ in output like:

0.7ε≤Δ≤1.43ε.

The upper boundary was chosen such that the geometric mean of both bounds is ε. A change of 1% of initial concentration in the near equilibrium regime will therefore lead to a change of 0.7–1.43 % of maximum air concentration. It should be noted that Equation (41) is only necessary for substance concentration and is not a prerequisite for other parameters.

The approximate solution for small concentrations and the solution for pure substance have a large overlap regarding the sensitive parameters. Differences occur regarding the molecular weight of matrices compared to the molecular weight of the substance, and the concentration plays no role in case of pure substance.

In case of the quick release regime, the vapor pressure only plays a role for the time it takes to reach the maximum air concentration, which is due to its comparatively short high values. Therefore, room air concentration dynamics are quite independent from vapor pressure. On the other hand, the amount of substance only influences the time for the concentration decrease for the near equilibrium and the ventilation driven regime, which depending on the chosen exposure time may not affect mean event concentration.

It is also possible to study the sensitivity if we take relationships between parameters into account, e.g., one can assume that surface area and total amount of product/substance are linear dependent, if the amount of product per surface area is constant. Increasing surface area under these circumstances will increase maximum substance concentration in the air for the quick release as well as for the ventilation driven regime. For the near equilibrium regime, the maximum substance concentration in air is not affected. Regarding the time scale of increasing substance air concentration, it will only be affected in the near equilibrium regime. There is no significant dependence in the quick release case, since dependence on surface area will be offset by increasing product amount, which has the opposite effect on the time duration for increase. Additionally, the time scale for decrease (respective the plateau stage for pure substance) is only affected in case of the near equilibrium regime and will increase with increasing surface area, given that product amount increases too.

### 3.4. Regime Graph

The mentioned three border cases or regimes are an abstraction, while any real situation will be a mixture of all three. A useful way to classify any scenario is to attribute weights for each regime. Let w be the weight, and the sub-indices A, B, C refer to the quick release, the near equilibrium, and the ventilation driven regime, respectively. Recalling Equation (16):

$$b=\frac{K\;S}{X_{0}}\frac{M_{r}}{RT}\frac{P_{vap}}{\left(\frac{1}{c_{0}}-1\right)}+\frac{K\;S}{V}+Q,$$

the weights w can be defined such that they reflect their relative contribution to the term b:

(42) $$w_{A}=\frac{\frac{K\;S}{X_{0}}\frac{M_{r}}{RT}\frac{P_{vap}}{\left(\frac{1}{c_{0}}-1\right)}}{\frac{K\;S}{X_{0}}\frac{M_{r}}{RT}\frac{P_{vap}}{\left(\frac{1}{c_{0}}-1\right)}+\frac{K\;S}{V}+Q}$$

(43) $$w_{B}=\frac{\frac{K\;S}{V}}{\frac{K\;S}{X_{0}}\frac{M_{r}}{RT}\frac{P_{vap}}{\left(\frac{1}{c_{0}}-1\right)}+\frac{K\;S}{V}+Q}$$

(44) $$w_{C}=\frac{Q}{\frac{K\;S}{X_{0}}\frac{M_{r}}{RT}\frac{P_{vap}}{\left(\frac{1}{c_{0}}-1\right)}+\frac{K\;S}{V}+Q}.$$

For illustration purpose, we will introduce a two-dimensional graphical method that visualizes to which (if any) regime the respective scenario belongs. For the x axis, the following value is assigned:

(45) $$u_{l}=\frac{\frac{K\;S}{V}}{\frac{K\;S}{V}+\frac{K\;S}{X_{0}}\frac{M_{r}}{RT}\frac{P_{vap}}{\left(\frac{1}{c_{0}}-1\right)}}=\frac{1}{1+\frac{V\;M_{r}}{X_{0}\;RT}\frac{P_{vap}}{\left(\frac{1}{c_{0}}-1\right)}}.$$

For the y-axis, the following value is used:

(46) $$v_{l}=\frac{Q}{\frac{K\;S}{V}+\frac{K\;S}{X_{0}}\frac{M_{r}}{RT}\frac{P_{vap}}{\left(\frac{1}{c_{0}}-1\right)}+Q}.$$

$u_{l}$ and $v_{l}$ range both from 0 to 1. The meaning of the index “l” will be explained later in this section.

**Figure 3:**
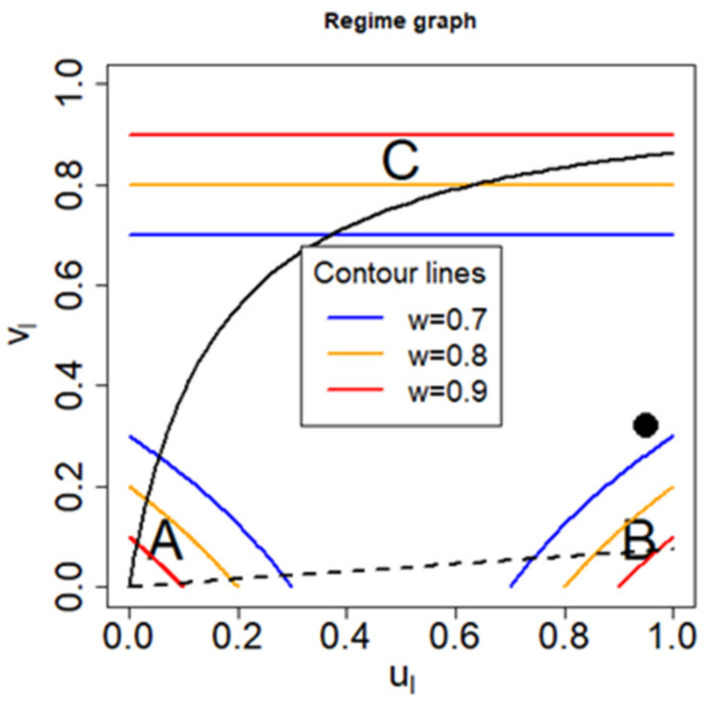
General regime graph depicting the position of the A) quick release, B) near equilibrium, and C) ventilation driven regime. Some contour lines for the weights of each regime are depicted. The black fat point at the right indicates a specified (arbitrary) scenario. The black curves show all possible positions in the regime graph for two respective scenarios from the ConsExpo paint products factsheet: the solid curve presents the line for the brush- and roller-painting–two-component paints–mixing and loading, while the dashed curve shows the line for the water borne wall paint scenario.

The value of u_l_ on the x-axis depicts where the situation between the quick release and the near equilibrium regime is located, while the $v_{l}$ value on the y-axis describes how relevant the ventilation rate Q is. We will refer to this graphical method as “regime graph”. Figure 3 shows the position of the three regimes together with the contour lines of the respective weights. The bold dot shows as an example the position of an arbitrarily chosen scenario. Although it cannot be clearly assigned to one regime, it can be seen that it tends to be close to a near equilibrium case, but has a significant influence of the ventilation driven regime.

As mentioned in the introduction, the National Institute for Public Health and the Environment (RIVM) in the Netherlands has published several fact sheets for a variety of consumer uses, which consist of all the parameters necessary for an exposure assessment, except substance related parameters such as substance concentration in the product, molecular weight, and vapor pressure. We use this information to study whether the regime graph can lead to insights given a specific scenario of such a fact sheet without knowledge of the substance, especially if it can enable risk assessors to narrow down for each of these scenarios which of the three regimes (quick release, near equilibrium, ventilation driven) are actually possible.

We can express *v*_l_ in terms of *u*_l_ (Equations (45) and (46)) and arrive at

(47) $$v_{l}=\frac{u_{l}}{u_{l}+\frac{K\;S}{V\;Q}}.$$

This shows that the parameters specified by the ConsExpo factsheets already limit possible positions in the regime graph to the curve defined above. Since u_l_ is maximal one, the term KS/VQ determines the maximal impact of ventilation regarding the influence on the respective regime, e.g., whether the ventilation driven regime can be feasible or not. Therefore, the line of possible positions in the regime graph is fixed by the term KS/VQ provided by the ConsExpo factsheets. While the calculation of u_l_ requires the validity of the approximation for small concentrations, the curve

$$v_{l}=f(u_{l})$$

itself can be generalized, since it does not depend on the approximation (hence the index l can be dropped). Figure 3 shows the corresponding curves for two scenarios, which are described in the ConsExpo paint products factsheet [12], namely for the brush- and roller-painting–two component paints–mixing and loading in solid line and for the water borne wall paint scenario in dashed line. For the former one, it can be seen that either the ventilation driven regime, the quick release regime, or a mixture of both regimes are possible, depending on the substance used. However, the exposure cannot be in the near equilibrium regime. For the water borne wall paint scenario, it can be observed that either the quick release regime, the near equilibrium regime, or a mixture between both are possible, but not the ventilation driven regime.

From the mentioned three parameters that are not fixed in the factsheets and needed to determine u_l_, the molecular weight of substance is not necessary for sufficient small concentrations. For small concentrations, we can use the following approximation:

(48) $$X_{0}\left(\frac{1}{c_{0}}-1\right)\approx \frac{X_{0}}{c_{0}}=A_{tot},$$

but the constraint (Equation (41)) should not be violated even for qualitative conclusions:

$$c_{0}\leq 0.3.$$

Hence,

(49) $$u_{l}=\frac{1}{1+\frac{V\;M_{r}}{X_{0}\;RT}\frac{P_{vap}}{\left(\frac{1}{c_{0}}-1\right)}}\approx \frac{1}{1+\frac{V\;M_{r}\;P_{vap}}{A_{tot}\;RT}}$$

(50) $$v_{l}=\frac{Q}{\frac{K\;S}{V}+\frac{K\;S}{X_{0}}\frac{M_{r}}{RT}\frac{P_{vap}}{\left(\frac{1}{c_{0}}-1\right)}+Q}\approx \frac{Q}{\frac{K\;S}{V}+\frac{K\;S}{A_{tot}}\frac{M_{r}}{RT}P_{vap}+Q}.$$

This means that for the assumption of small substance concentration, the actual value of this concentration plays approximately no role for determining *u*_l_ and $v_{l}$ for small concentrations, given that total product amount $A_{tot}$ is reported in the scenario descriptions. The actual position on the curve on the regime graph depends therefore solely on the vapor pressure of the substance. For sufficiently large vapor pressure ($u_{l}$ near 0), this line always ends in the lower left corner, which indicates the quick release regime.

Until here, all the considerations regarding the regime graph relied on the assumption

$$\frac{M}{M_{r}}\left(\frac{1}{c_{0}}-1\right)\gg 1.$$

However, if this assumption is not really met any longer, how will it affect the position on the regime graph? Therefore, it is useful to return to Equation (37), which is also a Taylor expansion of the evaporation term, but performed at the beginning of the evaporation process. We can now similarly set up terms for $u_{r}$ and $v_{r}$ that will describe the position on the regime graph for the alternative approximation (detailed analytical derivation given in the Supplementary Materials Part A1.3) and arrive at:

(51) $$u_{r}=\frac{1}{1+\frac{V\;M}{X_{0}\;RT}\;\frac{P_{vap}\;\frac{M}{M_{r}}\left(\frac{1}{c_{0}}-1\right)}{{\left(1+\frac{M}{M_{r}}\left(\frac{1}{c_{0}}-1\right)\right)}^{2}}}.$$

Accordingly, the new y-coordinate $v_{r}$ can be calculated using Equation (47). It should be noted that $u_{r}$ > $u_{l}$ and likewise $v_{r}$ > $v_{l}$. The index l therefore refers to “left” and the index r to “right” in order to label their position on the regime graph relative to each other.

Now for a given exposure scenario, a substance with defined concentration in the product can be represented by two points on the regime graph, where once the x-coordinate is calculated by using Equation (45) ($u_{l}$, $v_{l}$) and once by using Equation (51) (*u*_r_, $v_{r}$). The real situation at the start of the evaporation process will be represented by (*u*_r_, $v_{r}$), while during the evaporation process it will move towards ($u_{l}$, $v_{l}$). If both points still tend towards the same regime, the dependencies shown in Table 1 can be still used qualitatively to get a rough understanding of the dependencies of the parameters, although Equation (40) might be violated. However, in such cases, it is advised only to rely on those parameters in Table 1 that are listed for both the small concentration approximation and the pure substance case.

$$\frac{M}{M_{r}}\left(\frac{1}{c_{0}}-1\right)\geq 2.5,$$

**Figure 4:**
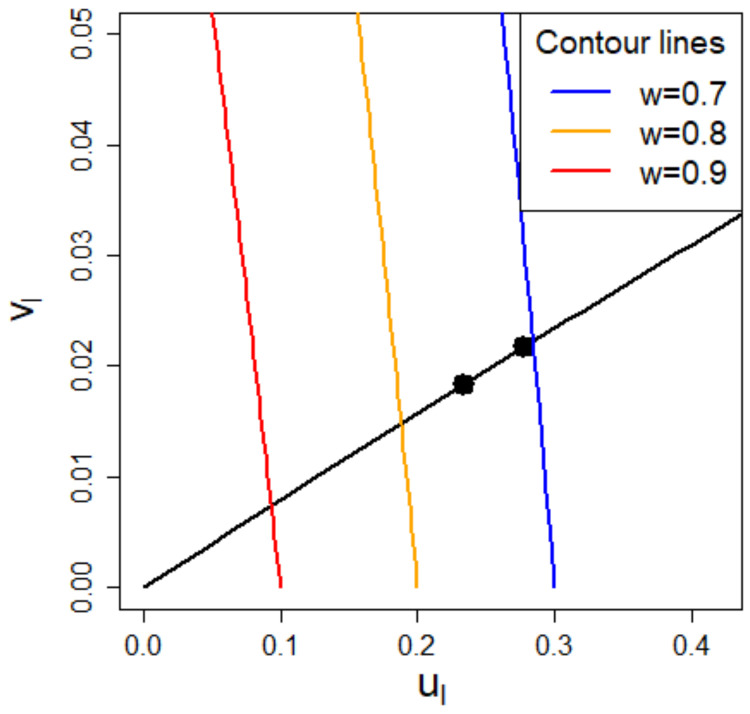
A segment of the regime graph is depicted in the area of the quick release regime. The solid line indicates all possible positions of any substance in the water borne wall paint scenario. The right and left point belong to a substance with vapor pressure of 10,000 Pa, a molecular weight of 80 g/mol, and initial concentration in the product of 20%. The right point represents the situation at the start of the evaporation process, but the situation moves down along the curve, while the substance evaporates until it reaches the left point.

In Figure 4, for a substance with vapor pressure of 10,000 Pa, a molecular weight of 80 g/mol, and initial concentration in the product of 20% the coordinates ($u_{l}$, $v_{l}$), and (*u*_r_, $v_{r}$) are depicted for the water borne wall paint scenario. We get:

$$\frac{M}{M_{r}}\left(\frac{1}{c_{0}}-1\right)=2.67.$$

The evaporation process starts at the right point and moves down along the specified scenario curve due to evaporation of the substance in the product until the left point is reached. In the Supplementary Materials Part B, a short practical guide is presented on how to get started with classifying an exposure situation towards the evaporation regimes and how to draw the regime graph.

### 3.5. Case Study for the Water Borne Wall Paint Scenario

In a previous project at the German Federal Institute for Risk Assessment (BfR), substances found in paint strippers were identified. Solvent-based paint strippers can cause serious injuries including respiratory irritation, narcosis, and allergic reactions through accidental inhalation of vapors or skin contact [23]. The exposure of consumers to many of the chemicals used in paint strippers is not well studied, which is why the ongoing project at BfR evaluates information from REACH (Registration, Evaluation, Authorization, and Restriction of Chemicals) registration dossiers for these substances. REACH (EC 1907/2006) forces manufacturers and importers of chemical substances within the European Union to disclose information about the substance and its hazards and conduct an exposure and risk assessment in the registration dossier. Substances used in paint strippers (e.g., hydrocarbon solvents) may also be used in wall paints. Therefore, we inferred that the water borne wall paint scenario (defined in the ConsExpo paint products factsheet [12]) is a plausible scenario to estimate consumer exposure.

The parameters fixed for the water borne wall paint scenario are listed in the Supplementary Materials Part C. The corresponding curve on the regime graph for this scenario is shown in Figure 3 in dashed line. We can conclude that either the near equilibrium regime, the quick release regime, or a mixture of both regimes apply to this scenario.

**Figure 5:**
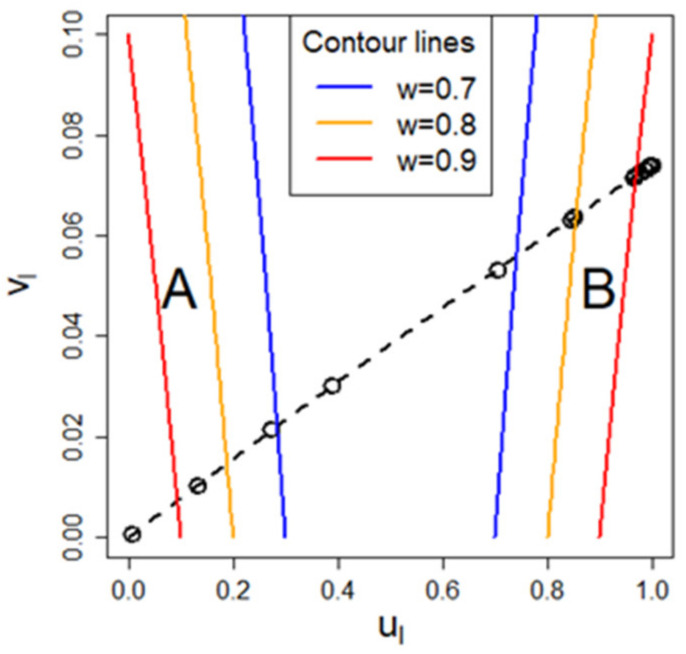
A segment of the regime graph is shown. The dashed line indicates the possible positions of any substance for the water borne wall paint scenario in the regime graph. In total, the positions of 18 substances are represented by empty circles. (**A**) denotes the quick release regime, while (**B**) denotes the near equilibrium regime. Contour lines for both regimes are shown too.

Within the ongoing project about substances found in paint strippers, 18 substances were evaluated, which are listed in the Supplementary Materials Part C together with their respective vapor pressure at 20 °C (the data for the vapor pressure were collected from databases and online resources such as PubChem (PubChem, Bethesda, MD, USA; https://pubchem.ncbi.nlm.nih.gov (accessed on 03 July 2020)), ECHA’s registered substances factsheets (ECHA, Helsinki, Finland; https://echa.europa.eu/search-for-chemicals (accessed on 03 July 2020)), and GESTIS substance database (IFA, Berlin, Germany; www.dguv.de/ifa/gestis-database (accessed on 03 July 2020)). Assuming sufficiently small concentrations, the position of each substance on the regime graph can be determined (shown in Figure 5 as points by using Equations (49) and (50)). Many points are located on the right side, indicating the near equilibrium regime. If we adopt a loose definition that any point on the regime graph belongs to a regime with a respective weight of no less than 0.7, 16 out of 18 substances can be attributed to a regime (three to the quick release and 13 to the near equilibrium regime). A stricter definition to a respective weight of no less than 0.8 still yields still 13 out of 18 successful classifications. 

The sensitive parameters and possible risk management measures for those substances that can clearly be assigned to a single regime can be extracted from Table 1. For the 13 substances in the near equilibrium regime, the control of substance concentration, for example, significantly influences the peak concentration. However, since the time for declining concentrations is rather long, increasing volume or increasing ventilation rate might not be effective for reducing mean event concentration for typical exposure times. In contrast, for three substances in the quick release regime, peak concentration would be affected by limiting the total amount of substance or also by increasing room volume as long as the treated surface area remains constant. Increasing ventilation considerably shortens the declining time of substance room concentration, and therefore influences the mean event concentration. Finally, there are two substances right in between the quick release and near equilibrium regime. We can still use Table 1 to obtain the most sensitive parameters by identifying those parameters that are listed as sensitive for both regimes. As long as the total product amount remains constant, substance concentration would be the most sensitive parameter for peak concentration. Regarding the timescale for declining substance concentration in room air, the ventilation rate would be the most sensitive parameter.

### 3.6. Influence of the Mass Transfer Coefficient on Maximum Concentration

The value of the mass transfer coefficient is associated with large uncertainty, with different models estimating a range of 2–16 m/h (ConsExpo default: 10 m/h), which shows the substantial uncertainty regarding the value of the mass transfer coefficient. In the following, we are going to study the influence of this uncertainty on maximal air concentration. From Table 1, we can see that in the quick release and near equilibrium regime, the mass transfer coefficient does not affect maximum air concentration in contrast to the ventilation driven regime. Therefore, if we analyze this problem using the regime graph, the larger $v_{l}$, the larger will changes of the mass transfer coefficient affect maximum air concentration. By Equation (47), $v_{l}$ is given by

$$v_{l}=\frac{u_{l}}{u_{l}+\frac{K\;S}{V\;Q}}.$$

If *u*_l_ is close to one (substances with very small vapor pressure), $v_{l}$ will be maximized. In this case, the assumption of small concentrations in Equation (10) is not needed, and the following results are valid for all concentration values. In the following, the two scenarios depicted in Figure 3 (the waterborne wall paint scenario and the brush- and roller painting–two component paints–mixing and loading scenario) are considered with a mass transfer coefficient of K = 10 m/h. The former scenario is for $u_{l}$ = 1 located in the near equilibrium regime, the latter scenario in the ventilation driven regime. In the following, we are studying the relative change in maximum air concentration for K_1_ = 2 m/h and 16 m/h compared to the standard value of K_2_ = 10 m/h. Using Equations (13)–(16) and comparable small vapor pressure *P_vap_*, the following can be inferred for the ratio r of maximum air concentrations:

(52) $$r:=\frac{y_{max}\left(K_{1}\right)}{y_{max}\left(K_{2}\right)}=\frac{K_{1}}{K_{2}}\frac{K_{2}\frac{S}{V}+Q}{K_{1}\frac{S}{V}+Q}.$$

Regarding the waterborne wall paint scenario and for K = 2 m/h, we get *r* = 0.77, which means that although K was reduced by a factor five, maximum air concentration has only reduced by a factor of 1.3. Using for the same scenario K = 16 m/h or a 60% increase, we yield for *r* = 1.03, or a modest 3% increase in maximum air concentration. At least for the water borne wall paint scenario, the large uncertainty of the mass transfer coefficient does not really affect maximum air concentration. However, the case of the brush-and roller-painting–two component paints–mixing and loading scenario is different. If K is reduced by a factor five to K = 2 m/h, we get *r* = 0.22, a 4.5 times smaller maximum air concentration than with K = 10 m/h. For K = 16 m/h or a 60% increase, we get *r* = 1.48 or a 48% increase in maximum air concentration. These results show that for this scenario, the uncertainty of the mass transfer coefficient K can have a decisive effect on maximum air concentration. The more a scenario is influenced by the ventilation driven regime, the more sensitive it is towards changes of the mass transfer coefficient.

In general, criteria can be defined to classify whether a scenario might be sensitive to the mass transfer coefficient K or not. If the maximum concentration is not allowed to drop by more than 33.3% (a factor 1.5), while K reduces from 10 to 2 m/h, we can use Equation (52) to derive the condition:

$$\frac{S}{V}\geq 0.7\;Q.$$

Given the data for an unspecified room used in ConsExpo (General Factsheet) (V = 20 m^3^, Q = 0.6/h), this would mean a surface area of at least 8.4 m^2^. A more tolerant criterion that allows the maximum concentration to drop by 50% (a factor 2) would yield:

$$\frac{S}{V}\geq 0.3\;Q,$$

resulting for the unspecified room in a minimum surface area of 3.6 m^2^. Such criteria can be applied to quickly scan the scenarios outlined in the ConsExpo fact sheets in order to filter those that might be vulnerable to changes of mass transfer coefficients. Finally, it is important to keep in mind that such scenarios do not necessarily need to be vulnerable if substances are used with sufficiently large vapor pressures.

## 4. Discussion

### 4.1. Benefits of the Numerical Approach

We have derived an approximate analytical solution for the set of differential equations used in the ConsExpo Web model for evaporation targeting small concentration ranges. It was demonstrated that this approximate solution captures the qualitative behavior of the system very well for parameter ranges that occur in many realistic assessments. The analytical solution allowed the identification of three boundary cases or regimes, with each case having its distinctive characteristics. These regimes are not limited to the domain of small concentrations, but they also occurred in the analytical solution for pure substance. The analytical expressions derived for different regimes allowed a simple sensitivity analysis, in which the important parameters were obtained.

Three different evaporation regimes were identified: the quick release regime, the near equilibrium regime, and the ventilation driven regime. Each regime features its own characteristics, and for each regime, sensitive parameters can be identified. This leads to three benefits: First, for a given scenario, it becomes clear for which parameter efforts should be undertaken to reduce uncertainty. Second, it helps to understand under which circumstances uncertainties have a substantial outcome on the exposure assessment, as demonstrated for the case of the mass transfer coefficient. Third, it is beneficial to understand which risk management options are promising. The proposed approach would be to look first at the influential parameters for the respective regime as the first candidates for effective risk management measures. For example, limiting the concentration itself may not help much in the quick release case, as long as total product amount is not kept constant. Similarly, when dealing with the near equilibrium regime, increasing the ventilation rate may not be very effective, depending on exposure time. Of course, if the ventilation rate is increased strongly enough, the evaporation regime will change to the ventilation driven regime and maximum air concentration decrease. However, the point is that the factor by which the ventilation rate needs to be increased is much larger than the factor by which maximum air concentration is decreased, and therefore (depending on the circumstances) it may be not very effective.

Apart from these results, the analytical solution also provided some further interesting findings. For small concentrations, it was found that the evaporation is influenced by the molecular weight of the matrix of the product and not the molecular weight of the substance. Additionally, for small substance concentrations, the evaporation regime does not change if the concentration of the substance changes. Consequently, it is not substance amount but product amount that determines whether an exposure situation belongs to the quick release regime or not. Finally, for small substance concentrations and pure substance alike, the formula for determining the time scale for increase of room air concentration of the quick release regime is the same as for the time scale for decrease of room air concentration in the ventilation driven regime. The same holds true for the respective other time scale with decreasing room air concentration for the quick release and increasing for the ventilation driven regime. This implies for the ventilation driven regime that the ventilation rate influences the time needed until maximum air concentration is reached, and vapor pressure (among other factors) influences the time needed to remove the substance from the room.

It is important to note that the aim of the approximate analytical solution is not to replace the original differential equations of ConsExpo. The numerical model will always be superior to the approximate solution. However, the analytical results are well suited to supplement the numerical model, because the problem with relying solely on numerical models is that no matter how many different simulations are run with them, at best only a modest amount of general knowledge can be gained by it, since each new set of new parameters represents a new situation. It is due to the approximate analytical approach that we can confidently infer about the existence of regimes.

One important advantage of the classification into regimes is that it provides a useful vocabulary to communicate the actual findings: “The exposure event is close to the near equilibrium regime, no wonder changing product amount does not much alter the maximum air concentration” is much more useful than “although I keep changing the product amount, the maximum air concentration does not alter much.” The approximate analytical solution allows thinking about results and consequently gaining an understanding of the underlying model.

The simple knowledge of towards which regime the exposure situation tends already comes with a set of valuable information of the dominant parameters listed in Table 1. For example, having a couple of chemical safety reports of REACH registrants with varying product amounts does not require much attention from the risk assessor if the exposure situation tends to be in the near equilibrium regime. While it is undeniable that a full fletched global sensitivity analysis will produce more accurate results, it would also require much more effort. Especially, if many substances are to be considered for a given use-scenario, the simplicity of the approach presented here will be very advantageous and discussed in the next subsection.

### 4.2. Benefits of the Regime Graph

For categorizing exposure scenarios, we have developed the regime graph. It shows to which degree the scenario tends to a regime or between which regimes it is located. This information in turn allows identifying the sensitive parameters using Table 1. We found that for any given ConsExpo scenario (defined by the respective factsheets), a defined curve exists on which all substances are located and that therefore limits the set of possible regimes. The curve itself, in contrast to the position on the curve, does not depend on the assumption of small concentrations and is therefore a more general feature of the system. Especially for small concentrations, we found that only the vapor pressure of the substance plays a role to determine the position on the line, since other parameters either are given by the ConsExpo Factsheet or play no substantial role.

The regime graph is very suitable to study many substances for the same use. Not only can risk assessors get a very quick overview about the dominant regimes, but this graphical method also is suitable for a grouping approach by grouping those substances together that belong to the same evaporation regime and are therefore susceptible to the same risk measures. We expect that a use-driven risk assessment (the study of many chemicals for a given use) will gain importance in risk assessment. One important reason is to avoid regrettable substitutions, and therefore it will become necessary to study groups of substances related to a common use. In legal procedures such as the restrictions of chemicals, it already occurs that groups of chemicals are considered. One example is the risk management option analysis (RMOA) in a total of ten solvents (https://echa.europa.eu/de/rmoa/-/dislist/details/0b0236e184ff4bdb (accessed on 02 July 2020)), which are grouped together not primarily because of similar toxicological concerns, but because of their function as solvents. We expect that for such assessments, the regime graph will be a very helpful tool.

### 4.3. Comparison with Previous Published Results

Jayjock [10] studied evaporation in the case of pure substance and unlimited substance amount. Using two scenarios for a given room and chemical substance, a small area was used one time and a large area another time. By varying the ventilation rate, he found that unlike the case of small surface area, the peak concentration for the large surface was not sensitive to changes of the ventilation rate. Our research does confirm these findings, given that the small surface scenario corresponds to the ventilation driven regime, while the large surface scenario corresponds to the near equilibrium regime. Moreover, we could expand on the results of Jayjock [10], since we defined clear criteria for the occurrence of each regime, generalized the results to the case that the substance is only a fraction of the product, and finally took the limited substance amount into account.

Arnold and Ramachandran [20] performed a sensitivity analysis of the instantaneous release model of ConsExpo. Utilizing four scenarios (nail polish scenario, bath water scenario, hand soap scenario, and carpet cleaner scenario), for each scenario the parameters and its uncertainty were defined. The authors employed 10,000 Monte Carlo simulations, estimated the total inhaled exposure, and calculated the respective standardized regression coefficients. They concluded that across the four scenarios, product amount, concentration, ventilation rate, and exposure time are the most important parameters. Any comparison with the research outlined needs to bear in mind that the results of Arnold and Ramachandran are strongly influenced by the uncertainty ranges assigned to the parameters. However, the instantaneous release model can be viewed as a simplification of the quick release regime. The influence of ventilation rate, exposure time, and total substance amount (which includes product amount and substance concentration listed as important parameters in [20]) can also be confirmed for the quick release regime. Actually, the analytical solution provides us with the information not to treat product amount and concentration separately, but include it as total substance amount. Finally, we want to emphasize that the evaporation process cannot be limited to the quick release regime (or instantaneous release model), since two qualitative distinctive regimes, the near equilibrium regime and the ventilation driven regime, can also occur.

### 4.4. Assumptions and Limitations of the Used Approach

The research showcased in this paper employed the differential equations used in ConsExpo Web (which were in turn derived from [9,10]) for describing the evaporation process as a starting point. Hence, they rely on all the assumptions and simplifications of the original model. One main assumption is the validity of the ideal gas law. Another issue is the assumption of an ideal mixture, such that the vapor pressure of a substance in the mixture is equal to the vapor pressure of the pure substance multiplied with its mole fraction (Raoult’s law). This actually neglects the cohesive and adhesive forces between different chemicals in a mixture. Furthermore, the evaporation model of ConsExpo Web assumes that all chemicals in the mixture apart from the substance in question are non-volatile and do not evaporate. Nevertheless, this model employs the main features of the evaporation process: a source term dependent on vapor pressure of the substance and its concentration in the product; the backpressure of the substance in the air; and the removal of substance in the air via ventilation. As a result, we expect that the evaporation model of ConsExpo is able to describe the crucial features of evaporation.

The approximate analytical solution of the set of differential equations relied on the assumption of small concentrations, whereby the interpretation of “small” is modified by the ratio of molecular weight of the matrix and the substance, respectively. If the molecular weight of the matrix is several times larger than the one of the substance, it will shrink the applicability domain of the approximation to smaller concentrations. Given that the approximate analytical solution is not applicable for every practical case, we have set clear criteria under which circumstances its use will be beneficial. Moreover, we developed another approximate analytical solution that deviates from the original model in the opposite direction and therefore allows the estimation of the impact of the error made. Finally, for the position of any exposure situation on the regime graph, we proposed to use a two-point system, one representing the situation at the beginning of the evaporation process and the other one doing the same for the end of the evaporation. During the time course, the system will move from the first point to the second one.

In this regard, we want to mention that a couple of results of this article actually do not depend on the assumption of small concentrations. Firstly, the existence of the three evaporation regimes is an overall feature of the evaporation process, as shown for the case of pure substance. Secondly, for a specified scenario setting, leaving only substance specific information such as molecular weight (of substance and matrix), vapor pressure, and concentration open, all possible cases are located on a defined curve in the regime graph. Thirdly, the analysis presented in this article under which circumstances the uncertainty associated with the mass transfer coefficient might affect exposure substantially does also not require the assumption of small substance concentration in the product.

Finally, we assumed instantaneous application of the product. While this assumption may indeed lead to significantly different exposure values, we expect that it does not really alter the qualitative nature of the evaporation process. Since in ConsExpo, the application rate of the product is constant, substance air concentration will peak only after the application finishes. The assumption of the instantaneous application will especially affect the time needed until maximum air concentration is reached. Furthermore, peak concentration might be smaller if application duration is large enough such that the ventilation rate can remove substantial parts of total substance outside the room. However, these facts do not change the qualitative nature of evaporation. Hence, the assumption of instantaneous application simplifies the model from complex mathematics, but leaves the evaporation process itself intact.

### 4.5. Analytical Solutions to Supplement Numerical Sensitivity Analysis

This article has presented an example of how the analytical solution can contribute to study parameter sensitivity. However, a much more common way of doing this is the use of numerical sensitivity analysis, e.g., variance based sensitivity analysis [21]. By defining the range and specific distribution for each parameter, the contribution to overall variance of the output can be obtained. One important advantage of this approach is that it treats the model itself as a “black box”, since the model dynamics and their underlying mathematics are ignored and only the numerical simulation runs are evaluated.

However, this advantage also turns out to be a disadvantage. The results of a numerical sensitivity analysis are only valid for the defined parameter ranges and therefore cannot be generalized. In addition they constitute a kind of averaging over this parameter space and thus do not allow inferring the sensitivity of parameters for a subspace of the studied parameter space. As presented in the analysis of this article, model behavior can lead to different regimes for certain parameter areas. For example, the maximum substance concentration is sensitive to changes of the ventilation rate in the ventilation driven regime, but not in the quick release and near equilibrium regime. Is the ventilation rate a sensitive parameter or not? It depends. Numerical sensitivity analyses with different parameter ranges will yield different results, but they cannot be generalized without further investigations. On the other hand, analyzing the analytical solution allows the identification of different areas (what we have in this article called “regimes”) of model behavior.

Not viewing models as black boxes, but analyzing the underlying mathematical equations can complement traditional sensitivity analyses and help paint a coherent picture of parameter sensitivity. We therefore think that using this approach is worthwhile when studying parameter sensitivity for other models. Regarding exposure models, the spray model of ConsExpo Web (exposure to spray: spraying) comes to mind [6]. This model has recently gained wider attraction due to comparison of its performance with experimental results [24,25]. An analytical solution for aerosol concentration is available given a defined aerosol diameter. The importance, e.g., of the ventilation rate on exposure will depend on other parameters, since aerosols are not only removed from room air via ventilation, but also via gravitational settling (falling to the floor), which in turn is influenced by the aerosol diameter. Further investigations might be therefore beneficial.

## 5. Conclusions

Obtaining an approximate analytical solution for small concentrations of the ConsExpo Web evaporation model brings new opportunities compared to a solely numerical treatment of the problem. In detail, the analytical solution allows a deeper understanding of the underlying dynamics, resulting in the identification of three qualitatively different regimes. Matching the relevant scenario to one of these regimes leads to a quick way to roughly know the sensitive parameters, allowing prioritizing the important parameters and finding promising risk management measures. The introduction of the regime graph, a graphical method to depict the position of a given scenario compared to the three regimes, is especially beneficial when dealing with many substances related to the same use. In this situation, the regime graph provides not only a quick overview of the overall situation, but also allows grouping of substances according to the dominant regime.

## Figures and Tables

**Table 1:** List of sensitive parameters according to the chosen variable and boundary case. (+) denotes linear (positive) dependence, (−) denotes an inverse relationship. Cells that are divided list the parameters on the left for the small concentration approximation, while the right one is for pure substance. The lowest row refers to the time for decrease in case of the small concentration approximation and the plateau stage for pure substance (except for the quick release, where the plateau stage does not exist).

Characteristic of Concentration Profile	Quick Release		Near Equilibrium		Ventilation Driven	
		
Maximal air concentration	X_0_ (+)V (−)		P_vap_ (+)		P_vap_ (+) V (−) Q (−) K (+) S (+)	
			c_0_ (+)M_r_ (+)	M (+)	c_0_ (+)M_r_ (+)	M (+)
Time for increase	P_vap_ (−) K (−) S (−) A_tot_ (+)		V (+) K (−) S (−)		Q (−)	
	M_r_ (−)	M (−)				
Time for decrease/plateau stage	Q (−)		P_vap_ (−) V (−) Q (−) A_tot_ (+)		P_vap_ (−) K (−) S (−) A_tot_ (+)	
			M_r_ (−)	M (−)	M_r_ (−)	M (−)

## Data Availability

The data presented in this study are available in the Supplementary Material.

## Supplementary Materials

The following are available online at https://www.mdpi.com/1660-4601/18/6/2829/s1. Part A: Analytical derivations, Part B: A quick guide to get started with determining evaporation regimes and drawing regime graphs (including Table S1 Symbols and units of all necessary parameters and constants), Part C: Parameter values used for figures (including Table S2 Parameters given in the respective ConsExpo factsheets (employed for Figure 3, Figure 4, and Figure 5) as well as the ones used for Figure 1, Table S3 Parameters used for Figure 2 for the assumption of small substance concentrations, Table S4 Parameters used for Figure 2 for pure substance, Substances along with their vapor pressure at 20 °C used for Figure 5), Part D: Programming code.
