# Supplementary material for: Insights Gained from an Approximate Analytical Solution of the Evaporation Model Used by ConsExpo Web
Source: Int J Environ Res Public Health. 2021 Mar 10;18(6):2829. doi: 10.3390/ijerph18062829 (PMC8000556; doi:10.3390/ijerph18062829)
Supplement: Supplementary file 1 [file ijerph-18-02829-s001.pdf]

International Journal of
Environmental Research
and Public Health

MDPI

Supplemental Materials

Part A – Analytical derivations

A.1 Approximate analytical solution for small concentrations

We start here at Eq. 12 and show the detailed derivation of the analytical solution:

$$\frac{dY}{dt} = K\,S\,\frac{M_r}{RT}\,P_{vap}\,\frac{1-\frac{Y}{X_0}-\frac{Z}{X_0}}{\left(\frac{1}{c_0}-1\right)} - \frac{K\,S}{V}Y - QY$$

$$\frac{dZ}{dt} = Q\,Y \quad . \tag{S1}$$

We rewrite the amount of substance in room air in the form

$$\frac{dY}{dt} = a - bY - \frac{a}{X_0}Z \tag{S2}$$

with the parameters a and b

$$a = K\,S\frac{M_r}{RT}\frac{P_{vap}}{\left(\frac{1}{c_0}-1\right)} \tag{S3}$$

$$b = \frac{K\,S}{X_0}\frac{M_r}{RT}\frac{P_{vap}}{\left(\frac{1}{c_0}-1\right)} + \frac{K\,S}{V} + Q \quad . \tag{S4}$$

Differentiating Eq. S2 in respect to time and inserting dZ/dt from Eq. S1 yields

$$\ddot{Y} = -b\dot{Y} - \frac{a\,Q}{X_0}Y \tag{S5}$$

with the initial conditions

$$X(t=0) = X_0 \tag{S6}$$

$$Y(t=0) = 0 \tag{S7}$$

$$\frac{dY}{dt}\Big|_{t=0} = a \tag{S8}$$

$$Z(t=0) = 0 \quad . \tag{S9}$$

Using the typical approach for a homogeneous linear differential equation:

$$Y(t) = de^{-\lambda t} \tag{S10}$$

yields for the eigenvalues λ:

$$\lambda^2 = b\lambda - \frac{a\,Q}{X_0}$$

$$\lambda_{1,2} = \frac{b}{2}\left(1 \pm \sqrt{1 - \frac{4\,a\,Q}{X_0 b^2}}\right) .$$

That results in

$$Y(t) = d_1 e^{-\lambda_1 t} + d_2 e^{-\lambda_2 t} \quad . \tag{S11}$$

Using the initial conditions Eq. S7 and Eq. S8:

$$d_1 + d_2 = 0 \rightarrow d_1 = -d_2 \tag{S12}$$

$$-\lambda_1 d_1 - \lambda_2 d_2 = a \rightarrow d_2 = \frac{a}{\lambda_1 - \lambda_2} = -d_1 \ , \tag{S13}$$

which gives

$$Y(t) = \frac{a}{\lambda_1 - \lambda_2}\left(e^{-\lambda_2 t} - e^{-\lambda_1 t}\right) \ . \tag{S14}$$

The term $a/(\lambda_2-\lambda_1)$ describes an upper boundary for the maximal amount of substance in the room air (since the difference of the two exponential terms cannot be larger than 1), while λ_1 characterises the rate for increase of the substance in room air and the λ_2 characterises the rate for the decrease of substance in room air.

To understand the dynamics described by Eq. S14, we will investigate the term b (Eq. S4) and introduce b_1 and b_2:

$$b = \frac{K\,S}{X_0}\frac{M_r}{RT}\frac{P_{vap}}{\left(\frac{1}{c_0}-1\right)} + \frac{K\,S}{V} + Q = b_1 + b_2 + Q.$$

From Eq. S2 it appears that the term b is responsible for slowing down the increase of substance in room air, which leads first to the formation of the maximum and subsequently to the decrease of substance air concentration (while in Eq. S2 there is still an additional term Z a/X_0, it should be noted that a/X_0 is part of b, more precisely b_1 (see also Eq. S15)). The first term b_1

$$b_1 := \frac{K\,S}{X_0}\frac{M_r}{RT}\frac{P_{vap}}{\left(\frac{1}{c_0}-1\right)} = \frac{a}{X_0} \tag{S15}$$

results from decreasing concentration of the substance in the product which decreases evaporation of that substance into room air. The term b_2

$$b_2 := \frac{K\,S}{V} \tag{S16}$$

results from the vapour pressure of the substance in room air. Finally, Q describes the transport from the substance in room air outside via ventilation. If we consider in Eq. S14 the denominator $\lambda_1-\lambda_2$,

$$\lambda_1 - \lambda_2 = b\sqrt{1 - \frac{4\,a\,Q}{X_0 b^2}} \ . \tag{S17}$$

Let us consider the following asymptotic cases (hereinafter referred to "regimes"):

1. $b_1 \gg b_2 + Q$; The initial amount of substance in the product limits maximum amount of substance in room air.

2. $b_2 \gg b_1 + Q$; The vapour pressure of the substance in the air (backpressure) limits the maximum amount of substance in room air.

3. $Q \gg b_1 + b_2$; Ventilation is dominant in limiting maximum amount of substance in room air.

For these three regimes, the rates for substance increase and decrease, λ_1 and λ_2, respectively, can be simplified to

$$\lambda_1 \approx b - \frac{a\,Q}{X_0 b} \approx b \quad \text{(S18)}$$

$$\lambda_2 \approx \frac{a\,Q}{X_0 b} \quad \text{(S19)}$$

$$\lambda_1 - \lambda_2 \approx b - \frac{2\,a\,Q}{X_0 b} \approx b\ . \quad \text{(S20)}$$

The following simplified analytical expressions can be derived for each regime (from here on the substance concentration y=Y/V in room air is used).

A1.1 Quick release regime

$$y(t) \approx \frac{X_0}{V}\left(e^{-\lambda_2 t} - e^{-\lambda_1 t}\right) \quad \text{(S21)}$$

With using $(1/c_0\text{-}1) \approx 1/c_0$ and $X_0/c_0 = A_{tot}$,

$$\lambda_1 \approx \frac{K\,S}{X_0}\frac{M_r}{RT}\frac{P_{vap}}{\left(\frac{1}{c_0} - 1\right)} \approx \frac{K\,S}{A_{tot}}\frac{M_r}{RT}P_{vap} \quad \text{(S22}$$

$$\lambda_2 \approx Q \quad \text{(S23)}$$

A1.2 Near equilibrium regime

$$y(t) \approx \frac{M_r}{RT}\frac{P_{vap}}{\left(\frac{1}{c_0} - 1\right)}\left(e^{-\lambda_2 t} - e^{-\lambda_1 t}\right) \quad \text{(S24)}$$

$$\lambda_1 \approx \frac{K\,S}{V} \quad \text{(S25)}$$

$$\lambda_2 \approx \frac{V}{X_0}\frac{M_r}{RT}\frac{P_{vap}}{\left(\frac{1}{c_0} - 1\right)}Q \approx \frac{V}{A_{tot}}\frac{M_r}{RT}Q\,P_{vap} \quad \text{(S26)}$$

A1.3 Ventilation driven regime

$$y(t) \approx \frac{K\,S}{Q\,V}\frac{M_r}{RT}\frac{P_{vap}}{\left(\frac{1}{c_0} - 1\right)}\left(e^{-\lambda_2 t} - e^{-\lambda_1 t}\right) \quad \text{(S27)}$$

$$\lambda_1 \approx Q \quad \text{(S28)}$$

$$\lambda_2 \approx \frac{K\,S}{X_0}\frac{M_r}{RT}\frac{P_{vap}}{\left(\frac{1}{c_0} - 1\right)} \approx \frac{K\,S}{A_{tot}}\frac{M_r}{RT}P_{vap} \quad \text{(S29)}$$

A1.2 Analytical solution for pure substance

As we have derived an approximate analytical solution for small concentrations, we compare it with the solution for pure substance. Pure substance implies c_0 equal to one; therefore, the following term disappears:

$$\frac{M}{M_r}\left(\frac{1}{c_0}-1\right)=0\ . \qquad \text{(S30)}$$

The equilibrium vapour pressure then simply reads:

$$P_{eq}=P_{vap}\ .$$

The set of differential equations outlined in Eq.5 (of the original article) are reduced to:

$$\frac{dX}{dt}=-K\,S\frac{M}{RT}P_{vap}+K\frac{S}{V}Y;X(t)>0 \text{ otherwise } \frac{dX}{dt}=0$$

$$\frac{dY}{dt}=K\,S\frac{M}{RT}P_{vap}-K\frac{S}{V}Y-Q\,Y \quad \text{for any t with } X(t)>0$$

$$\frac{dY}{dt}=-Q\,Y \quad \text{for any t with } X(t)=0 \qquad \text{(S31)}$$

These differential equations are linear and solved according to the approach used in subsection 2.2. Let t^{end} be the time until the amount of substance in liquid form has completely evaporated, then the solution reads (y=Y/V):

$$y(t)=\frac{K\frac{S\,M}{VRT}P_{vap}}{K\frac{S}{V}+Q}\left(1-e^{-\left(K\frac{S}{V}+Q\right)t}\right);t\le t^{end} \qquad \text{(S32)}$$

$$y(t)=\left[\frac{K\frac{S\,M}{VRT}P_{vap}}{K\frac{S}{V}+Q}\left(1-e^{-\left(K\frac{S}{V}+Q\right)t^{*}}\right)\right]e^{-Q\left(t-t^{end}\right)};t\ge t^{end}\ . \qquad \text{(S33)}$$

There can be in principle three different periods for room air concentration dynamics: First, the increase in room air concentration; second, the plateau phase, where concentration practically remains constant; third, the decrease of room air concentration after all the substance has evaporated. The second stage does not occur in every case.

We want to mention that for the special case of unlimited substance supply, Eq. S32 was published already in (Jayjock 2010).

Apart from the case of pure substance, for large concentrations and larger molecular weight of the matrix compared to the substance, such that

$$\frac{M}{M_r}\left(\frac{1}{c_0}-1\right)\ll 1\ ,$$

the derived analytical solution might be a good approximation until most of the substance has evaporated from the product.

In analogue to the defined asymptotic cases or regimes for the assumption of small concentrations, we carry out a similar analysis here.

A1.2.1 Quick release regime

$$X_0 \ll \frac{K\,S\frac{M}{RT}P_{vap}}{K\frac{S}{V}+Q} \quad \text{(S34)}$$

Maximal room air concentration: X_0/V .

The time until the substance completely evaporates can be estimated by neglecting air vapour pressure, which yields according to the system of differential equations (Eq. S31) an evaporation rate of $K\,S\frac{M}{RT}P_{vap}$. Therefore, the time t^+ until the maximum of room concentration is reached is estimated to:

$$t^+ \approx \frac{X_0\,R\,T}{K\,S\,M\,P_{vap}} \;. \quad \text{(S35)}$$

In this regime, there is no plateau stage.

A1.2.2 Near equilibrium regime

$$X_0 \gg \frac{K\,S\frac{M}{RT}P_{vap}}{K\frac{S}{V}+Q} \text{ and } K\frac{S}{V} \gg Q \quad \text{(S36)}$$

Maximal room air concentration: $y \approx \frac{M}{RT}P_{vap}$. (S37)

Time scale for concentration increase in room air: $t^+ \approx \frac{V}{K\,S}$. (S38)

To estimate the time the system remains in the plateau stage, t*, we need to estimate the evaporation rate (Eq. S31) during the plateau stage (using maximal room concentration given in Eq. S33). When the maximum concentration is approximately reached, the substance evaporates with a rate of:

$$K\,S\frac{M}{RT}P_{vap} - K\,S\frac{K\,S\frac{M}{V\,RT}P_{vap}}{K\frac{S}{V}+Q} = K\,S\frac{M}{RT}P_{vap}\left(1-\frac{K\frac{S}{V}}{K\frac{S}{V}+Q}\right) \approx \frac{Q\,V\,M\,P_{vap}}{RT} \;.$$

The time until the substance completely evaporates, is therefore given by:

$$t^* \approx \frac{X_0\,RT}{Q\,V\,M\,P_{vap}} \;. \quad \text{(S39)}$$

In the near equilibrium regime, the maximal room air concentration will reach nearly the saturated vapour pressure; the evaporation rate is very small under such condition.

A1.2.3 Ventilation driven regime

$$X_0 \gg \frac{K\,S\frac{M}{RT}P_{vap}}{K\frac{S}{V}+Q} \text{ und } K\frac{S}{V} \ll Q \quad \text{(S40)}$$

Maximal room air concentration: $y \approx \frac{K\frac{S\,M}{V RT}P_{vap}}{Q}$ (S41)

Time scale for concentration increase in room air: $t^{+} \approx \frac{1}{Q}$ (S42)

When maximum concentration is approximately reached, the substance evaporates with a rate of

$$K\,S\,\frac{M}{RT}P_{vap} - K\,\frac{S}{V}\frac{K\,S\frac{M}{RT}P_{vap}}{Q} = K\,S\,\frac{M}{RT}P_{vap}\left(1-\frac{K\frac{S}{V}}{Q}\right) \approx K\,S\,\frac{M}{RT}P_{vap}\ .$$

Therefore, the time until the substance completely evaporates is given by:

$$t^{*} \approx \frac{X_0\,RT}{K\,S\,M\,P_{vap}} \qquad \text{(S43)}$$

In the ventilation driven regime, the maximal air concentration will be much lower than the saturated vapour pressure; the evaporation rate is therefore substantial.

A1.3 Alternative approximation

The alternative Taylor expansion, where no substantial part of the substance has evaporated yet (Eq. 37), reads:

$$P_{vap}\,\frac{1-\frac{Y}{X_0}-\frac{Z}{X_0}}{1-\frac{Y}{X_0}-\frac{Z}{X_0}+\frac{M}{M_r}\left(\frac{1}{c_0}-1\right)} \approx \frac{P_{vap}}{1+\frac{M}{M_r}\left(\frac{1}{c_0}-1\right)} - P_{vap}\,\frac{\frac{M}{M_r}\left(\frac{1}{c_0}-1\right)\left(\frac{Y}{X_0}+\frac{Z}{X_0}\right)}{\left(1+\frac{M}{M_r}\left(\frac{1}{c_0}-1\right)\right)^2} = P_{vap}\,\frac{1+\frac{M}{M_r}\left(\frac{1}{c_0}-1\right)\left(1-\frac{Y}{X_0}-\frac{Z}{X_0}\right)}{\left(1+\frac{M}{M_r}\left(\frac{1}{c_0}-1\right)\right)^2}\ .$$

Using this approximation would alter the term b (Eq. 16) to

$$b = \frac{K\,S}{X_0}\frac{M}{RT}\frac{P_{vap}\,\frac{M}{M_r}\left(\frac{1}{c_0}-1\right)}{\left(1+\frac{M}{M_r}\left(\frac{1}{c_0}-1\right)\right)^2} + \frac{K\,S}{V} + Q\ . \qquad \text{(S44)}$$

Subsequently the x-coordinate u_r reads (in analogy to Eq. 45):

$$u_r = \frac{\frac{K\,S}{V}}{\frac{K\,S}{V}+\frac{K\,S\,M}{X_0\,RT}\frac{P_{vap}\,\frac{M}{M_r}\left(\frac{1}{c_0}-1\right)}{\left(1+\frac{M}{M_r}\left(\frac{1}{c_0}-1\right)\right)^2}} = \frac{1}{1+\frac{V\,M}{X_0\,RT}\frac{P_{vap}\,\frac{M}{M_r}\left(\frac{1}{c_0}-1\right)}{\left(1+\frac{M}{M_r}\left(\frac{1}{c_0}-1\right)\right)^2}} \qquad \text{(S45)}$$

$$v_r = \frac{u_r}{u_r+\frac{K\,S}{V\,Q}}\ . \qquad \text{(S46)}$$

Part B – A quick guide to get started with determining evaporation regimes and drawing regime graphs

For practitioners who want to apply the findings of this article to their own work, but find this article a bit lengthy to pick the necessary technical steps, we want to present one (by no means mandatory) way to get started.

Table S1. Symbols and units of all necessary parameters and constants.

Symbol	Name	Unit (Value)
K	Mass transfer coefficient	m/h
S	Surface area	m^2
V	Room volume	m^3
M	Molecular weight of substance	kg/mol
M_r	Molecular weight of product matrix	kg/mol
R	Universal gas constant	8.314 J/(K mol)
T	Temperature	K (Kelvin)
A_0	Total product amount	kg
X_0	Total substance amount	kg
c_0	Initial substance concentration in the product	g/g
P_{vap}	Vapour pressure of the substance	Pa (Pascal)
Q	Ventilation rate	1/h

Case 1: Single substance, concentration unknown but assumed small

Use a simplification of Eq. 42-44 to determine

$w_A = \frac{\frac{K\,SM_r}{A_0\,RT}P_{vap}}{\frac{K\,SM_r}{A_0\,RT}P_{vap}+\frac{K\,S}{V}+Q}$ (weight for quick release regime)

$w_B = \frac{\frac{K\,S}{V}}{\frac{K\,SM_r}{A_0\,RT}P_{vap}+\frac{K\,S}{V}+Q}$ (weight for near equilibrium regime)

$w_C = \frac{Q}{\frac{K\,SM_r}{A_0\,RT}P_{vap}+\frac{K\,S}{V}+Q}$ (weight for ventilation driven regime).

If any of the w are larger than at least 0.7, a dominant regime can be identified and Table 1 be used to determine the dominant influencing parameters. Keep in mind that the result might not hold for larger concentrations, however if the result is a near equilibrium or ventilation driven regime

Case 2: Single substance, all information (including substance concentration in the product) are known

Step 1: Check if inequality (Eq. 40)

$\frac{M}{M_r}\left(\frac{1}{c_0}-1\right) \geq 2.5$

is met. If yes, proceed to Step 2. If not, consider Case 3A - 3E.

Step 2: Use Eq. 42 – 44 (the total substance amount X_0 can be calculated by $X_0 = A_0 * c_0$.) to determine

$$w_A = \frac{\frac{K\,SM_r\;P_{vap}}{X_0\,RT\left(\frac{1}{c_0}-1\right)}}{\frac{K\,SM_r\;P_{vap}}{X_0\,RT\left(\frac{1}{c_0}-1\right)}+\frac{K\,S}{V}+Q}$$ (weight for quick release regime)

$$w_B = \frac{\frac{K\,S}{V}}{\frac{K\,SM_r\;P_{vap}}{X_0\,RT\left(\frac{1}{c_0}-1\right)}+\frac{K\,S}{V}+Q}$$ (weight for near equilibrium regime)

$$w_C = \frac{Q}{\frac{K\,SM_r\;P_{vap}}{X_0\,RT\left(\frac{1}{c_0}-1\right)}+\frac{K\,S}{V}+Q}$$ (weight for ventilation driven regime).

If any of the w are larger than at least 0.7, a dominant regime can be identified and Table 1 be used to determine the dominant influencing parameters.

Case 3A: Regime graph – Drawing the contour lines

Let w be respective weight used for plotting the contour lines (choose e.g. w = 0.7, 0.8, and 0.9), then the following function need to be drawn for each value of w:

$v_l = 1 - \frac{w}{1-u_l}$; $0 \leq u_l \leq 1$-w; for the quick release regime

$v_l = 1 - \frac{w}{u_l}$; 1-w $\leq u_l \leq 1$; for the near equilibrium regime

$v_l = w$; $0 \leq u_l \leq 1$; for the ventilation driven regime

Plot u_l on the x-axis and v_l on the y-axis. These contour lines indicate the area for each regime.

Case 3B: Regime graph – Drawing of the scenario-related regime curve

For the exposure scenario in question, collect the parameters K, S, V, and Q, use Eq. 47

$$v = \frac{u}{u+\frac{K\,S}{V\,Q}} \;,$$

and plot v as a function of u with u running from 0 to 1 on the x-axis and v depicted on the y-axis. All substances must be on this curve. In certain cases, one regime might therefore be already excluded due to the location of this curve.

Case 3C: Regime graph – Plot position of substance(s) with unknown, but small concentration in the product

Use Eq. 49 and Eq. 47

$$u_l = \frac{1}{1+\frac{V\,M_r\,P_{vap}}{A_{tot}\,RT}}$$

$$v_l = \frac{u_l}{u_l+\frac{K\,S}{V\,Q}}$$

and plot u_l on the x-axis and v_l on the y-axis. Together with the contour lines of the respective regimes, it indicates the position of the specific exposure scenario towards the evaporation regimes. This process can be repeated for as many substances as desired. Keep in mind that the result might not hold for larger concentrations, however the real result would only move up along the regime curve, not down.

Case 3D: Regime graph – Plot position of substance(s) with known but sufficient small concentration in the product

Check if the inequality (Eq. 40)

$$\frac{M}{M_r}\left(\frac{1}{c_0}-1\right) \geq 2.5$$

is met. Otherwise, consider additionally Case 3E. Use Eq.45 and Eq. 47:

$$u_l = \frac{1}{1+\frac{V\,M_r}{X_0\,RT}\frac{P_{vap}}{\left(\frac{1}{c_0}-1\right)}}$$

$$v_l = \frac{u_l}{u_l+\frac{K\,S}{V\,Q}}$$

and plot u_l on the x-axis and v_l on the y-axis. Together with the contour lines of the respective regimes, it indicates the position of the specific exposure scenario towards the evaporation regimes. This process can be repeated for as many substances as desired.

Case 3E: Regime graph – Plot position of substance(s) with known but problematic substance concentration in the product

Use Eq. 51 and Eq. 47

$$u_r = \frac{1}{1+\frac{V\,M}{X_0\,RT}\frac{P_{vap}\frac{M}{M_r}\left(\frac{1}{c_0}-1\right)}{\left(1+\frac{M}{M_r}\left(\frac{1}{c_0}-1\right)\right)^2}}$$

$$v_r = \frac{u_r}{u_r+\frac{K\,S}{V\,Q}}$$

and plot u_r on the x-axis and v_r on the y-axis additionally to (u_l, v_l). Each substance is represented by two points. If both of those can be attributed to the same regime, Table 1 can be used for identifying important parameters.

Part C- Parameter values used for figures

For all numerical simulations, the value for the mass transfer coefficient K was chosen to be 10 m/h and a temperature T of 293.15 K was selected.

Table S2. Parameters given in the respective ConsExpo factsheets (employed for Fig. 3, Fig. 4, and Fig. 5) as well as the ones used for Fig. 1.

Parameter	Fig. 1	ConsExpo Paints Factsheet	ConsExpo Paints Factsheet
		Brush and roller painting; two-component paint; mixing and loading	Water borne wall paint; application
Product amount	100 g		3750 g
Concentration by weight	0.2 g/g		
Vapour pressure	40 Pa		
Molecular weight of substance	100 g/mol		
Molecular weight of matrix	100 g/mol	3000 g/mol	120 g/mol
Ventilation rate	2/h	0.6/h	0.6/h
Surface area	15 m^2	0.0095 m^2	15 m^2
Volume	20 m^3	1 m^3	20 m^3

Table S3. Parameters used for Fig.2 for the assumption of small substance concentrations.

Parameter	Quick release regime	Near equilibrium	Ventilation driven regime
Product amount	100 g	100 g	100 g
Concentration by weight	0.1 g/g	0.1 g/g	0.1 g/g
Vapour pressure	1000 Pa	10 Pa	10 Pa
Molecular weight of substance	100 g/mol	100 g/mol	100 g/mol
Molecular weight of matrix	100 g/mol	100 g/mol	100 g/mol
Ventilation rate	1/h	1/h	2.5/h
Surface area	15 m^2	15 m^2	1 m^2
Volume	20 m^3	20 m^3	20 m^3

Table S4. Parameters used for Fig.2 for pure substance.

Parameter	Quick release regime	Near equilibrium	Ventilation driven regime
Product amount	100 g	100 g	100 g
Concentration by weight	1 g/g	1 g/g	1 g/g
Vapour pressure	1000 Pa	40 Pa	100 Pa
Molecular weight of substance	100 g/mol	100 g/mol	100 g/mol
Molecular weight of matrix	100 g	100 g	100 g
Ventilation rate	1/h	1/h	2.5/h
Surface area	15 m^2	15 m^2	1 m^2
Volume	20 m^3	20 m^3	20 m^3

The vapour pressure shown in Table S5 were collected from databases and online resources such as PubChem (PubChem, Bethesda, MD, USA https://pubchem.ncbi.nlm.nih.gov), ECHA's registered substances factsheets (ECHA, Helsinki, Finland; https://echa.europa.eu/search-for-chemicals), and GESTIS substance database (IFA, Berlin, Germany; www.dguv.de/ifa/gestis-database).

Table S5. Substances along with their vapour pressure at 20°C used for Fig. 5.

Substance EC number	Substance name	Vapour pressure [Pa]
211-463-5	1,3-dioxolane	10132.47
220-250-6	1-ethylpyrrolidin-2-one	18.00
203-961-6	2-(2-butoxyethoxy)ethanol	2.70
500-241-6	isotridecanol, ethoxylated, 1-2.5 moles ethoxylated	0.01
931-254-9	hydrocarbons, c6, isoalkanes, <5% n-hexane	25000.00
204-812-8	sodium 2-ethylhexyl sulfate	1.20
204-626-7	4-hydroxy-4-methylpentan-2-one	129.00
225-878-4	1-butoxypropan-2-ol	139.00
203-933-3	2-butoxyethyl acetate	50.00
918-811-1	hydrocarbons, c10, aromatics, <1% naphthalene	90.00
927-510-4	hydrocarbons, c7, n-alkanes, isoalkanes, cyclics	6000.00
203-572-1	4-methyl-1,3-dioxolan-2-one	4.00
108-94-1	cyclohexanone	700
100-51-6	phenylmethanol	7
201-148-0	2-methylpropan-1-ol	1600.00

110-12-3	5-methylhexan-2-one	665.00
115-10-6	dimethyl ether	513289.70
201-114-5	triethyl phosphate	52.30

Part D – Programming code

The following R code calculates (and plots) substance air concentration for the ConsExpo Web model "Exposure to vapour: Evaporation" with instantaneous application und additionally determines (and plots) the two approximate analytical solutions discussed in 2.2 and 2.4.

```
# calculates numerical solution of the ConsExpo Web Exposure to vapour: evaporation model with
# instantaneous application

rm(list=ls())

dAA<-function(A1_prod,A1_air) {
  dAA<-array(0,2)
  if (A1_prod==0) {
    c1<-0
    dA1_prod<-0
    dA1_air<--Q*A1_air
  } else{
    c1<-A1_prod/(A1_prod+A2_prod)
    c2<-1-c1
    P1_eq<-P1_vap*c1/(c1+c2*M1/M2)
    P1_air<-A1_air*R*T/(V*M1)
    dA1_prod<--K*S*M1/(R*T)*(P1_eq-P1_air)
    dA1_air<-K*S*M1/(R*T)*(P1_eq-P1_air)-Q*A1_air
  }
  dAA[1]<-dA1_prod
  dAA[2]<-dA1_air
  dAA
}

A_prod<-0.1                 # total product amount in kg
cc1<-0.2                    # (mass) percentage of substance in product
cc2<-1-cc1
R<-8.3145                   # universal gas constant
T<-293.15                   # temperature in Kelvin
```

```
P1_vap<-40                # vapour pressure of substance in Pascal
K<-10/3600                # mass transfer coefficient expressed in m/s
S0<-15                    # (final) surface area in m^2
V<-20                     # room volume in m^3
M1<-0.1                   # molare mass of substance in kg
M2<-0.1                   # molare mass of product matrix in kg
Q<-2/3600                 # ventilation rate expressed in 1/s

Expo_time<-4*3600     # total exposure time in seconds


# initial conditions for time t=0

A1_prod_0<-A_prod*cc1 # initial substance amount in mixture
A1_air_0<-0               # initial substance amount in air
A2_prod_0<-A_prod*cc2 # initial product amount without substance

###################

dt<-1                     # time step in seconds
zeitschritte<-ceiling(Expo_time/dt) # number of time steps

A1_prod<-A1_prod_0
A1_air<-A1_air_0
A2_prod<-A2_prod_0
S<-S0
luft1<-array(0,zeitschritte)
zeit<-array(0,zeitschritte)

for (i in 1:zeitschritte) {
  k1<-dAA(A1_prod,A1_air)
  A1_prod_k2<-A1_prod+dt*k1[1]/2
  A1_air_k2<-A1_air+dt*k1[2]/2
  k2<-dAA(A1_prod_k2,A1_air_k2)
  A1_prod_k3<-A1_prod+dt*k2[1]/2
  A1_air_k3<-A1_air+dt*k2[2]/2
  k3<-dAA(A1_prod_k3,A1_air_k3)
  A1_prod_k4<-A1_prod+dt*k3[1]
  A1_air_k4<-A1_air+dt*k3[2]
  k4<-dAA(A1_prod_k4,A1_air_k4)
  A1_prod<-A1_prod+dt/6*(k1[1]+2*k2[1]+2*k3[1]+k4[1])
  A1_air<-A1_air+dt/6*(k1[2]+2*k2[2]+2*k3[2]+k4[2])
```

```
    if (A1_prod<0) {
      A1_air<-A1_air+A1_prod
      A1_prod<-0
    }
    luft1[i]<-A1_air
    zeit[i]<-i*dt
}
luft1<-luft1/V*1000000 # substance air concentration in mg/m^3 - numerical solution

Omega<-K*S0/(R*T)
gamma<-R*T/V
a<-Omega*M2*P1_vap/(1/cc1-1)
b<-a/A1_prod_0+Omega*gamma+Q

lambda1<--b/2+b/2*sqrt(1-4*Q*a/(b*b*A1_prod_0))
lambda2<--b/2-b/2*sqrt(1-4*Q*a/(b*b*A1_prod_0))


luft_ana<-a/(lambda1-lambda2)*(exp(lambda1*zeit)-exp(lambda2*zeit))
luft_ana<-luft_ana/V*1000000 # substance air concentration in mg/m^3 - main approximate
# analytical solution

aa<-Omega*M1*P1_vap/(1+M1/M2*(1/cc1-1))
bb<-aa/A1_prod_0+Omega*gamma+Q

lambda11<--bb/2+bb/2*sqrt(1-4*Q*aa/(bb*bb*A1_prod_0))
lambda22<--bb/2-bb/2*sqrt(1-4*Q*aa/(bb*bb*A1_prod_0))

luft_ana2<-aa/(lambda11-lambda22)*(exp(lambda11*zeit)-exp(lambda22*zeit))
luft_ana2<-luft_ana2/V*1000000    # substance air concentration in mg/m^3 - alternative
                                  # approximate analytical solution

zeit<-zeit/60 # time in minutes

par(mar=c(5,6,4,2)+0.1)

plot(zeit,luft1, col="black", type="l", xlim=c(0,240), ylim=c(0,230),xlab="Time in minutes", ylab = expression("Concentration in mg/m"^"3"),
      lwd=2, font.lab=1,cex.axis=1.6,cex.lab=2)
par(new=TRUE)
lines(zeit,luft_ana,col="black", lwd=2, lty=2)
par(new=TRUE)
```

```
lines(zeit,luft_ana2,col="black", lwd=2, lty=3)
legend("topright",legend=c("numerical solution","approximate solution 1","approximate solution 2"),
lty=c(1,2,3),lwd=2,cex=1.6)
```
